# Effects of Zinc Supplementation on the Incidence of Mortality in Preschool Children: A Meta-Analysis of Randomized Controlled Trials

**DOI:** 10.1371/journal.pone.0079998

**Published:** 2013-11-11

**Authors:** Wei Fu, Li-Ren Ding, Cheng Zhuang, Yu-Hao Zhou

**Affiliations:** 1 Department of Pediatrics, Shanghai Seventh People’s Hospital, Shanghai, China; 2 Department of Rehabilitation Institute, Shanghai Seventh People’s Hospital, Shanghai, China; Kyushu University Faculty of Medical Science, Japan

## Abstract

**Background:**

Previous trials have shown that zinc supplementation can decrease the risk of diarrhea, pneumonia, and malaria in children; however, the effects of zinc supplementation on mortality remain unclear. This study aimed at evaluating the benefits and risks of zinc supplementation on both total mortality and cause-specific mortality.

**Methodology and Principal Findings:**

We searched PubMed, EmBase, and the Cochrane Central Register of Controlled Trials to identify randomized controlled trials in preschool children reporting total mortality or cause-specific mortality. Relative risk (RR) was used as a measure of the effect of zinc supplementation on the risk of mortality using a random effect model. Of the 1,520 identified articles, we included 8 trials reporting data on 87,854 children. Overall, zinc supplementation had no effect on total mortality (RR, 0.76; 95% CI: 0.56–1.04; P = 0.084), diarrhea-related mortality (RR, 0.80; 95% CI: 0.53–1.20; P = 0.276), pneumonia-related mortality (RR, 0.52; 95% CI: 0.11–2.39; P = 0.399), malaria-related mortality (RR, 0.90; 95% CI: 0.77–1.06; P = 0.196), or other causes of mortality (RR, 0.98; 95% CI: 0.67–1.44; P = 0.917). Subgroup analysis indicated that zinc supplementation was associated with a reduction in total mortality risk if the participants were boys, aged greater than 12 months, and the duration of the follow-up period was less than 12 months.

**Conclusions/Significance:**

Zinc supplementation does not have an effect on total mortality, diarrhea-related mortality, pneumonia-related mortality, malaria-related mortality, or other causes of mortality. Subgroup analysis suggested that zinc supplementation can effectively reduce the risk of total mortality if the participants were boys, aged greater than 12 months, and the duration of the follow-up period was less than 12 months.

## Introduction

Zinc deficiency is common in children in developing countries due to the low food intake, restricted zinc bioavailability from local diets, and losses of zinc during diarrheal illness [1,2]. Over the past few decades, several studies [3,4] have shown that zinc supplementation had a beneficial impact on the incidence of diarrhea, pneumonia, and malaria and that it can increase weight gain among low-birth weight infants [5]. All of these are considered risk factors for mortality. Therefore, it has been suggested that increased plasma zinc may be able to prevent all-cause mortality; however, increased concentrations of zinc in the blood have not been shown to be consistently beneficial for mortality. 

Previous meta-analyses [6,7] indicated that zinc supplements may reduce the risk of diarrhea and pneumonia, but failed to improve total mortality, and cause-specific mortality. Although a slight survival advantage was detected for zinc supplementation, the difference was not statistically significant, which makes interpretation of these results difficult for clinicians and has further restricted its application in clinical prevention.

Zinc supplementation has been studied in several large-scale, randomized controlled trials [8-10]. To investigate zinc supplementation specifically and in greater detail, we carried out a systematic review and meta-analysis of pooled data from randomized controlled trials that evaluated the possible effects of zinc supplementation on total mortality and cause-specific mortality in preschool children. 

## Methods

### Search strategy and selection criteria

This review was conducted and reported according to the Preferred Reporting Items for Systematic Reviews and Meta-Analysis (PRISMA) Statement [11] issued in 2009 (Checklist S1). Randomized controlled trials of zinc supplementation, written in the English language, were eligible for inclusion in our meta-analysis, regardless of their publication status (published, in press, or in progress), and the effects of zinc supplementation on total mortality and cause-specific mortality in preschool children were examined. Relevant trials were identified using the following procedure:

1. Electronic searches: we searched the PubMed, EmBase, and Cochrane Central Register of Controlled Trials electronic databases for articles published through December 2012 using “zinc” AND “randomized controlled trials” AND “clinical trials” AND “human” as the search terms. All reference lists from reports on non-randomized controlled trials were searched manually for additional eligible studies.
2. Other sources: we searched ongoing randomized controlled trials in the metaRegister of Controlled Trials, which lists trials that are registered as completed but not published yet. Furthermore, we reviewed the bibliographies of publications for potentially relevant trials. Medical subject headings, methods, population, interventions, and outcomes variables of these studies were used to identify relevant trials.

The literature search, data extraction, and quality assessment were independently undertaken by 2 authors (WF and LRD) using a standardized approach. Any inconsistencies were settled by arbitration with the primary author (YHZ) until a consensus was reached. We restricted our research to randomized controlled trials, which were less likely than observational studies to be subject to confounding variables or bias. A study was deemed eligible for inclusion if the following criteria were met: (1) the study was a randomized controlled trial; (2) the number of events for mortality or cause-specific mortality that occurred during the study were reported by intervention and control groups; (3) participants were preschool children; (4) the trial evaluated and compared the effects of zinc supplementation and a placebo, and (5) the duration of follow-up was at least 6 months. 

### Data collection and quality assessment

Data extraction and quality assessment were conducted independently by 2 reviewers (WF, CZ) using a standardized extraction form. Information was examined and adjudicated independently by an additional author (YHZ) who referred to the original studies after data extraction and assessment. The following information was extracted from eligible trials: first author, year of publication, country, number of patients, percentage of boys, range of age, interventions, duration of follow-up, and rate of total mortality or cause-specific mortality for each group. One author (WF) entered the data into the computer, and another author (CZ) checked it. Any disagreement between the 2 authors was settled by discussion with a third author (YHZ) until a consensus was reached. Study quality was assessed using the Jadad score [12], which is based on the 5 following subscales: randomization (1 or 0), concealment of the treatment allocation (1 or 0), blinding (1 or 0), completeness of follow-up (1 or 0), and the use of intention-to-treat analysis (1 or 0). This generates a total score (ranging from 1 to 5) that has been developed for quality assessment. In our study, we considered any study given a score of 4 or above to be a high-quality study. 

### Statistical analysis

We allocated the results of each randomized controlled trial as dichotomous frequency data. The relative risks (RRs) and 95% confidence intervals (CIs) of the individual trials were calculated from the event numbers extracted from each trial before data pooling. The overall RRs and 95% CIs of total mortality and cause-specific mortality were also calculated. Both fixed-effect and random-effects models were used to assess the pooled RRs for zinc supplementation as compared to a placebo. Although both models yielded similar findings, results from the random-effects model presented here assume that the true underlying effect varies among included trials [13,14]. Furthermore, in the random-effects model, we used the prediction interval (PI) to illustrate the degree of heterogeneity in forests plots, which could also provide a predicted range for the true treatment effect in an individual study [15,16]. Heterogeneity of the treatment effects between studies was investigated visually using a scatter plot analysis and statistically using the heterogeneity I^2^ statistic [17,18]. We explored potential heterogeneity in estimates of the treatment effects using univariate meta-regression [19] (publication years, duration of follow-up). Subgroup analyses were conducted on the basis of publication years, country, sex, age, the duration of the follow-up period, and study quality. We also performed a sensitivity analysis by removing each individual trial from the meta-analysis. Visual inspection of funnel plots for incident stroke was conducted, and Egger [20] and Begg tests [21] were used to assess publication bias statistically and quantitatively. All P values were two-sided, and P values less than 0.05 were regarded as statistically significant for all tests. All statistical analyses were carried out using STATA software (version 10.0). 

## Results

**Figure 1:**
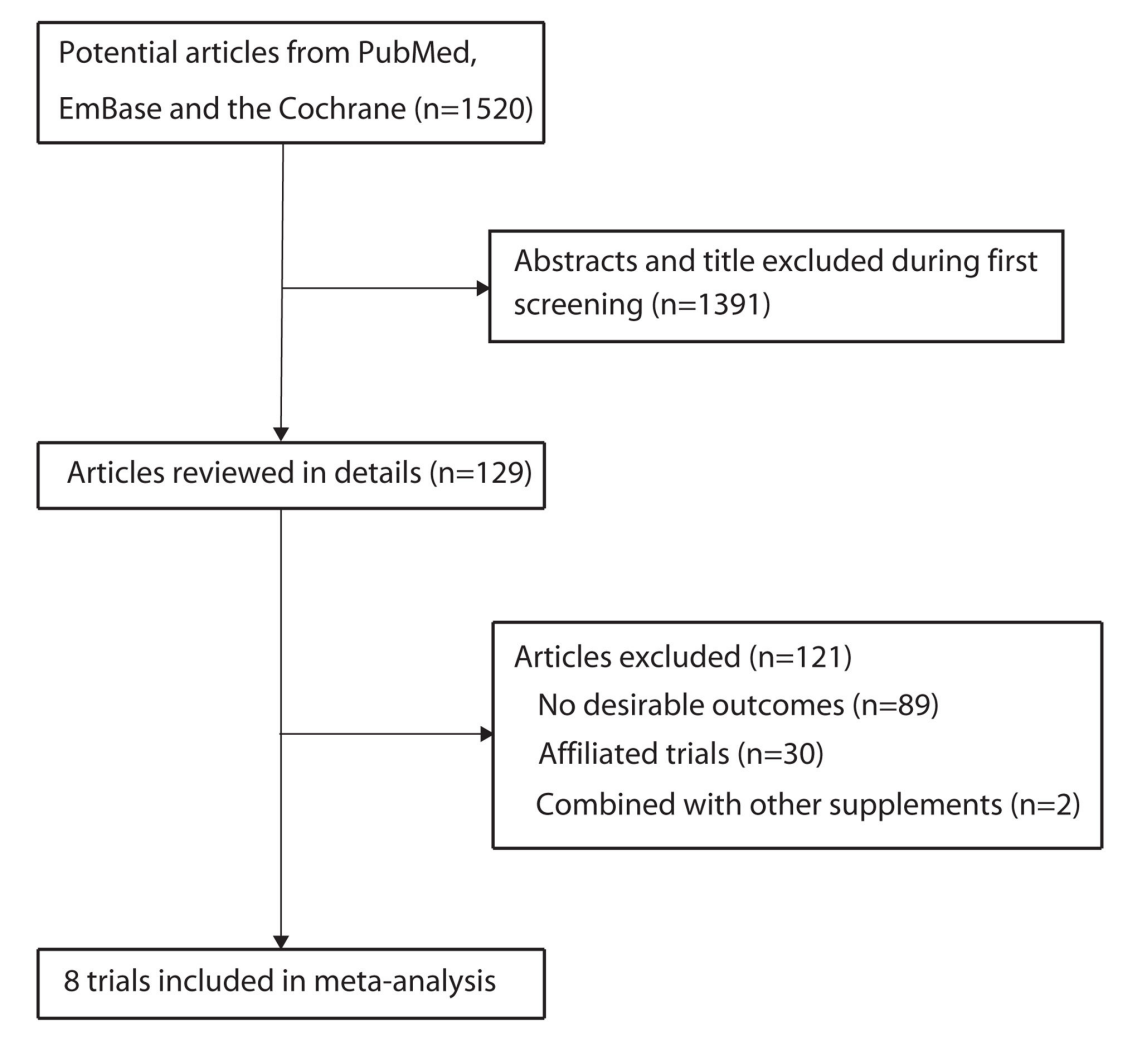
Flow diagram of the literature search and trials selection process.

We identified 1,520 articles during our initial electronic search, of which 1,391 were excluded following an initial review (title and abstract). We retrieved the full text for the remaining 129 articles, and 8 randomized controlled trials [8-10,22-26] were found to meet all of the inclusion criteria (Figure 1). Table 1 summarizes the characteristics of these trials and the important baseline information of the 87,854 individuals included. The trials included in this study compared the effects of zinc supplementation to that of a placebo for total mortality and cause-specific mortality. The population of the trials ranged from 96 to 42,546, and the follow-up for subjects ranged from 6 to 17.6 months. Although the included trials did not report on all of the key indicators of trial quality, the quality of the trials was assessed by the Jadad score. Overall, 5 trials [9,10,22,23,25] had a Jadad score of 4, 1 trial [8] had a score of 3, and the remaining 2 trials [24,26] had a score of 2.

**Table 1:** Design and characteristic of trials included in our meta-analysis.

**Source**	**Country**	**No. of subjects**	**Sex (boy, %)**	**Age (months)**	**Intervention**	**Follow-up (months)**	**Jadad score**
JM Tielsch 2007[8]	Nepal	41276	51.5	1-35	Zinc (10mg daily); placebo	17.6	3
WA Brooks 2005 [22]	Bangladesh	1621	51.9	2-11	Zinc (70mg weekly); placebo	11	4
KA Luabeya 2007 [23]	South Africa	226	53.1	4-6	Zinc (10mg daily); placebo	14.8	4
S Sazawal 2007[10]	Zanzibar	42546	50.4	1-36	Zinc (10mg daily); placebo	15.9	4
S Sazawal 2001[24]	India	1154	NG	1-9	Zinc (5mg daily); placebo	7	2
O Muller 2001[9]	Burkina Faso	661	49.2	6-31	Zinc (12.5mg daily); placebo	6	4
R Bobat 2005[25]	South Africa	96	49.0	6-60	Zinc (10mg daily); placebo	9	4
A Shankar 2000[26]	Papua new guinea	274	46.7	6-60	Zinc (10mg daily); placebo	10	2

**Figure 2:**
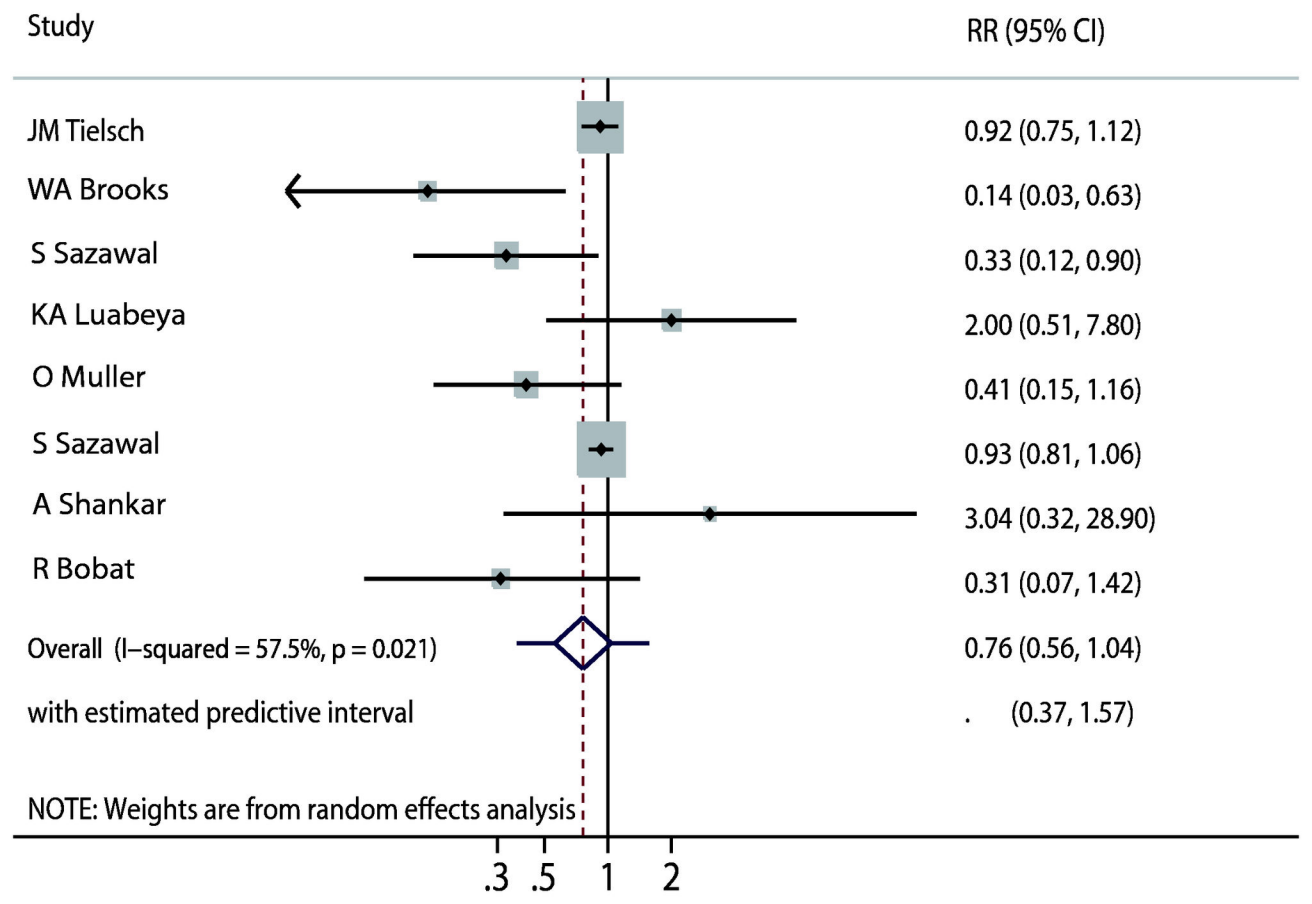
Effect of zinc supplementation on the risk of total mortality.

Data on the effect of zinc supplementation on total mortality were available from 8 trials [9,10,22,23,25], which included 87,854 children and reported 1,558 events of mortality. We noted that zinc supplementation showed a 24% reduction in total mortality; however, there was no supporting evidence to show that zinc supplementation protected against total mortality risk (RR, 0.76; 95%CI: 0.56–1.04, P = 0.084; 95%PI: 0.37–1.57; Figure 2). Heterogeneity was observed in the magnitude of the effect across the trials (I^2^ = 57.5%, P = 0.021). However, after sequential exclusion of each trial from all pooled analysis, the conclusion was not affected by the exclusion of any specific trial.

**Figure 3:**
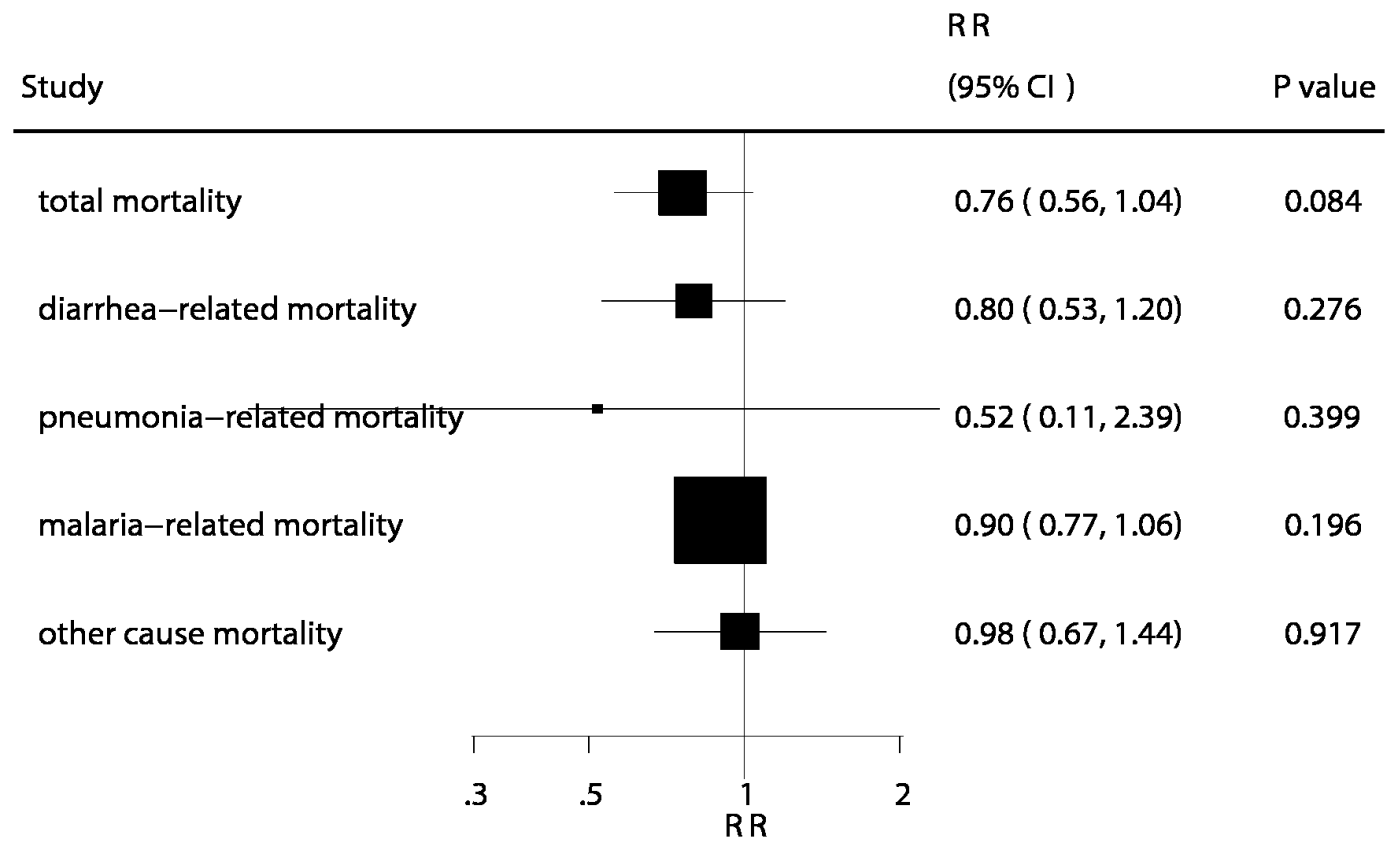
Summary of the relative risks of all outcomes assessed.

The number of trials available for each outcome were 4, 3, 1, and 3 for diarrhea-related mortality [8,10,22,24], pneumonia-related mortality [8,22,24], malaria-related mortality [10], and other causes of mortality [8,22,24], respectively. There were no differences observed between children receiving zinc and those receiving placebo for diarrhea-related mortality (RR, 0.80; 95% CI: 0.53–1.20; P = 0.276, Figure 3), pneumonia-related mortality (RR, 0.52; 95% CI: 0.11–2.39; P = 0.399, Figure 3), malaria-related mortality (RR, 0.90; 95% CI: 0.77–1.06; P = 0.196, Figure 3), or other causes of mortality (RR, 0.98; 95% CI: 0.67–1.44; P = 0.917, Figure 3). Unimportant heterogeneity was observed in the magnitude of the effect across the trials. A sensitivity analysis was also conducted, and after sequential exclusion of each trial from all pooled analysis, the conclusion was not affected by the exclusion of any specific trial. 

Substantial heterogeneity was observed in the magnitude of the effect for total mortality across the trials. We, therefore, conducted a meta-regression [19] analysis including publication years and duration of follow-up. However, these variables did not seem to be important factors contributing to the association between zinc supplementation and total mortality risk (publication years, P = 0.769; duration of follow-up, P = 0.732, Figure S1).

**Figure 4:**
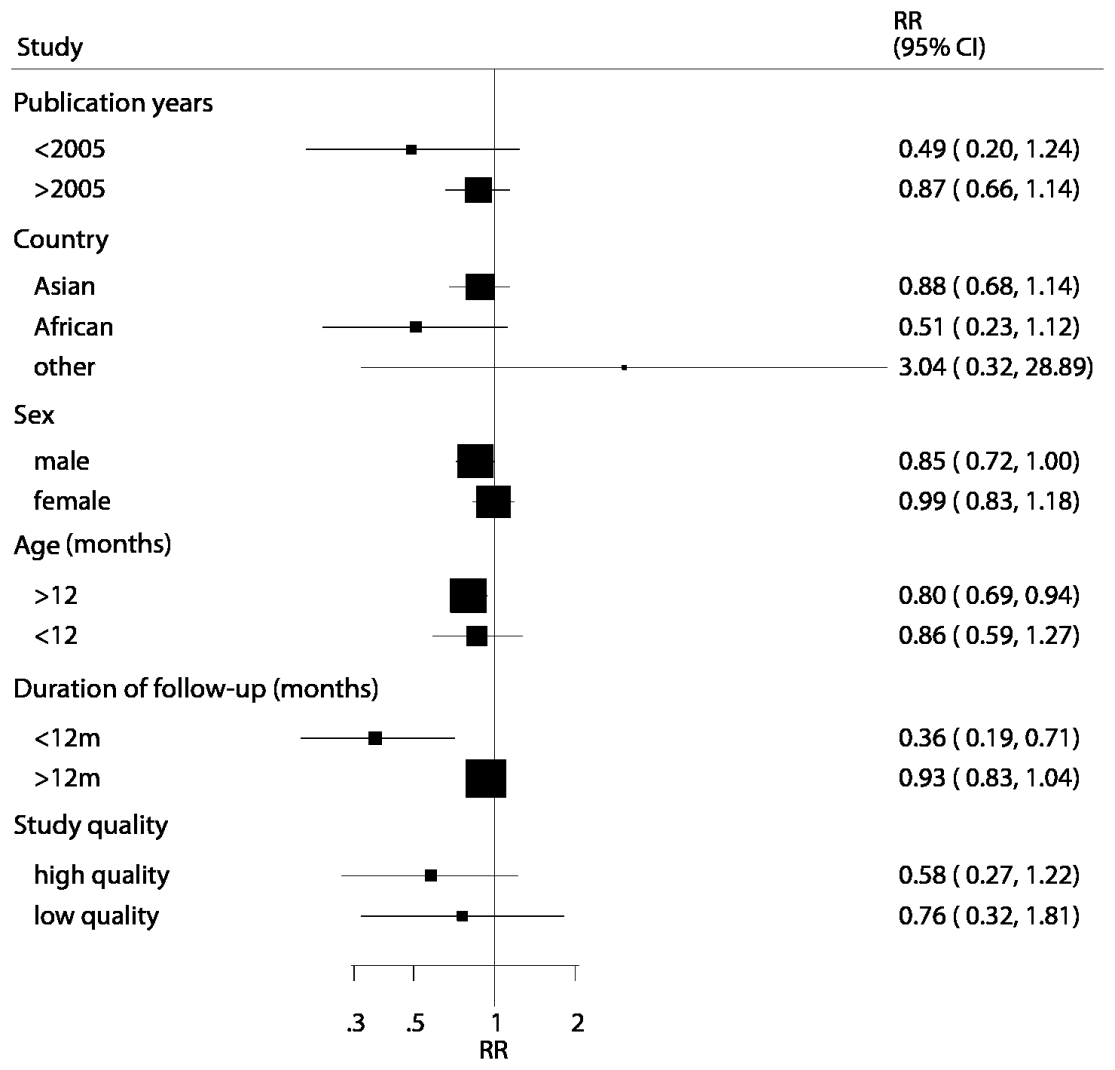
Subgroup analysis for total mortality.

Subgroup analyses were also conducted on tests for total mortality in order to minimize heterogeneity among included trials and to evaluate the effect of zinc supplementation in a specific population. We noted that zinc supplementation was associated with a reduction in total mortality if the participants were boys, aged more than 12 months, and the duration of the follow-up period was less than 12 months. No other significant differences were identified between the effects of zinc supplementation and placebo based on additional subset factors (Figure 4). 

**Figure 5:**
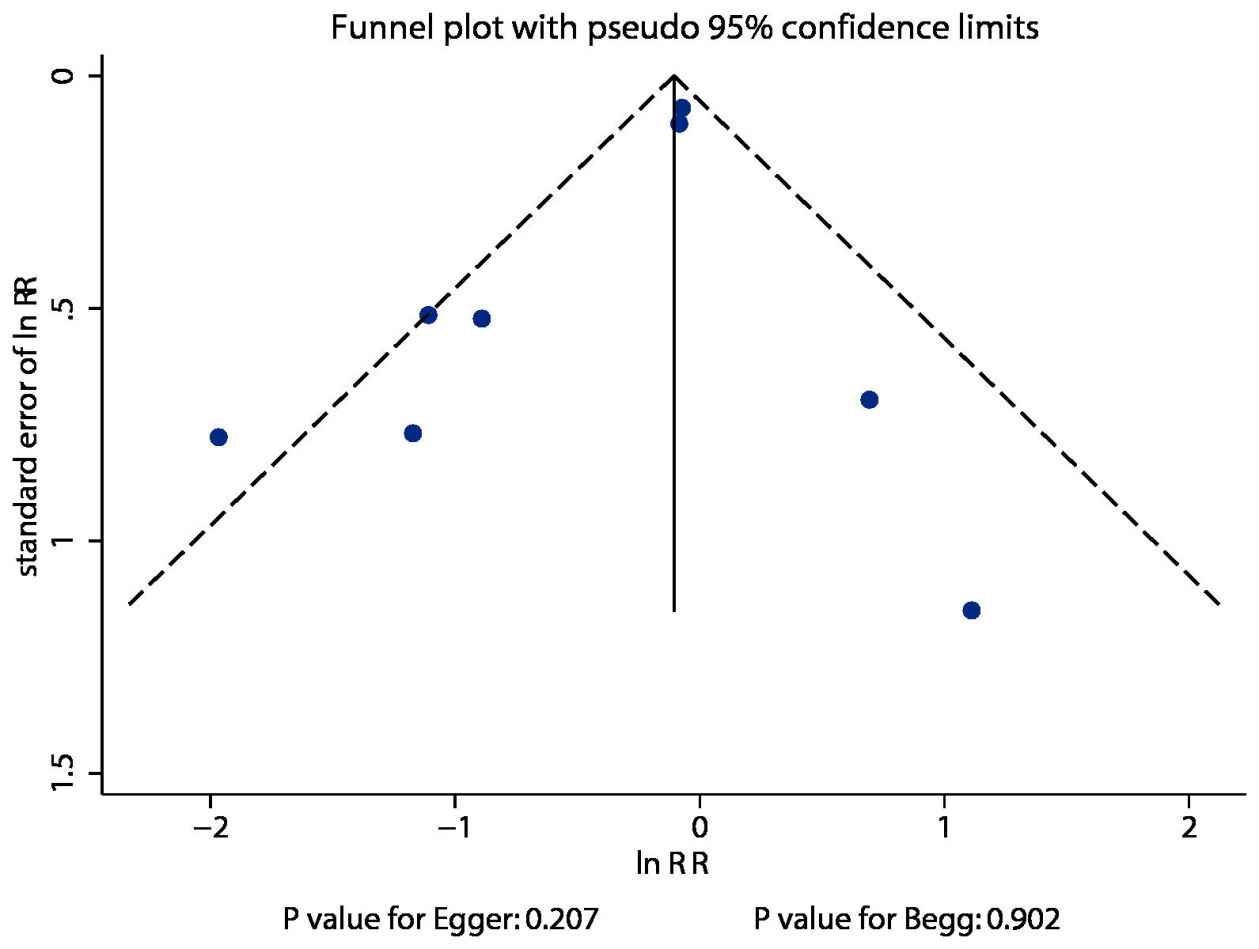
Funnel plot for total mortality.

Review of funnel plots could not rule out the potential for publication bias for total mortality (Figure 5). In addition, Egger [20] and Begg tests [21] showed no evidence of publication bias for total mortality (P value for Egger, 0.207; P value for Begg, 0.902). 

## Discussion

Zinc supplementation has a marked effect on the risk of diarrhea, pneumonia, and malaria that has already been described by randomized controlled trials [3-5] and meta-analysis [6]. However, the effect of zinc supplementation in reducing the risk of total mortality has not been confirmed by randomized controlled trials or meta-analysis. A previous meta-analysis [7] has evaluated the impact of zinc supplementation on mortality and morbidity due to diarrhea, pneumonia and malaria on the basis of randomized controlled trials. Although that meta-analysis [7] detected statistically significant reduction in diarrhea and pneumonia in trials that assessed the effects of zinc supplements, but failed to improve mortality, and cause-specific mortality. Our current study was based on randomized controlled trials and explored all possible correlations between zinc supplementation and the outcomes of total mortality and cause-specific mortality. This large quantitative study included 87,854 individuals from 8 trials with a broad range of baseline characteristics. The results of our meta-analysis suggest that zinc supplementation has no effect on the total mortality, diarrhea-related mortality, pneumonia-related mortality, malaria-related mortality, or other causes of mortality. 

Our main findings are consistent with the findings of previous meta-analysis [7], which concluded that zinc supplementation had no significant benefit or adverse effect on the risk of total mortality. The possible reasons for this lack of significant effect are as follows: (1) the use of background zinc supplementation might have impaired our ability to identify a treatment effect; (2) relatively few events of mortality were reported in several studies, which contributed to broad confidence intervals, and prevented us from obtaining an intrinsic effect; and (3) uncertain or missing causes of mortality occurs in developing country frequently, which could have reduced or balanced the effect of zinc supplementation on the risk of mortality. Therefore, although zinc supplementation may have direct beneficial effects on total mortality, these effects may be balanced by the above important factors. 

There was no significant difference between zinc supplementation and placebo for the relative risk of diarrhea-related mortality, pneumonia-related mortality, malaria-related mortality, or other causes of mortality. Although our study showed a protective effect in the risk of these outcomes, this difference was not statistically significant. The possible reasons for this lack of significant effects are as follows: (1) as the trials included were designed to evaluate the effects of zinc supplementation on cause-specific morbidity, but not mortality-related outcomes, these results were derived from very few cases and should be regarded as preliminary results; and (2) a smaller number of trials reported these outcomes, resulting in broader confidence intervals. 

In our current meta-analysis, subgroup analysis was also performed, which revealed that the risk of total mortality was significantly reduced if the participants were boys, aged greater than 12 months, and the duration of the follow-up period was less than 12 months. The possible reasons for this are follows (1). There are apparent differences in the effect of zinc supplementation by sex, with a benefit in boys and no benefit in girls, as zinc requirements for infant growth are higher for boys than for girls, which has been suggested as a possible reason for a greater effect in boys [27]. (2) There are apparent differences in the effect of zinc supplementation by age, with a benefit in individuals aged more than 12 months and no benefit in individuals aged less than 12 months, as infants may have received adequate zinc in utero [28] and are able to obtain adequate zinc from the breast milk, even though maternal stores are suboptimum [29]. Since breastfeeding rates were 95% or greater until the infant was aged 12 months in the study populations, we cannot compare the effects of zinc supplements between breastfeed and non-breastfeed infants. Alternatively, the absence of effects in this age group might be related to the low 5-mg dose used, compared with 10 mg or more in several studies, which could have balanced these treatment effects (3). The apparent differences in the effect of zinc supplementation by different duration of follow-up could possibly be due to chance, as fewer trials were included in this subset resulting in less variation of the conclusion.

The limitations of our study are as follows: (1) several trials may not have had a long enough duration to adequately identify the effects of zinc supplementation on mortality; (2) although subgroup analysis suggested zinc supplementation significantly reduced the risk of total mortality in some specific subsets, these results may be variable due to of the small number of trials that were included in these subsets; and (3) as inherent assumptions are made during any meta-analysis, the analysis used pooled data, and individual data was not available, we were restricted from performing a more detailed and relevant analysis and from obtaining more comprehensive results. 

The findings of this study suggest that zinc supplementation has no significant effects on total mortality, diarrhea-related mortality, pneumonia-related mortality, malaria-related mortality, or other causes of mortality. Subgroup analyses suggested that zinc supplementation significantly reduced the risk of total mortality of the participants who were boys, aged greater than 12 months, and the duration of the follow-up period was less than 12 months. Future studies should test a variety of interventions, including varying the dosage of supplementation, duration of supplementation, or giving the supplementation in combination with other important trace elements or multivitamins. We suggest that ongoing trials could be improved in the following ways: (1) any cause-specific mortality should be recorded and reported normatively, and it should be evaluated in any future trial; and (2) stratified analysis by sex, age, and other important factors should be taken into consideration before evaluating clinical outcomes. Through these, we might be able to confirm both the optimal time of supplementation and the optimal dosage of supplementation.

## Supporting Information

Checklist S1 **PRISMA Checklist.**
(DOC)

Figure S1 **Meta-regression of publication years and duration of follow-up for total mortality.**
(EPS)
